# Multigene phylogeny and taxonomy of Hydnaceae (Cantharellales, Basidiomycota) reveal *Leucopruina abieticola* gen. et sp. nov. from Southwest China

**DOI:** 10.3389/fmicb.2026.1889648

**Published:** 2026-08-14

**Authors:** Zhan-Bo Liu, Jian-Ling Zhang, Ren-Jin Chen, Ke-Ni Meng, Ying-Ge Fan, Han Liu, Guang-Peng Weng, Xiao-Bin Liu, Jing-Xuan Xu, Yu-Tong Zhang, Qing-Chao Zeng, Han Zhao, Shu-Jiang Li

**Affiliations:** 1 College of Forestry, Sichuan Agricultural University, Chengdu, China; 2 Forest Ecology and Conservation in the Upper Reaches of the Yangtze River Key Laboratory of Sichuan Province, Chengdu, China; 3 Sichuan Mt. Emei Forest Ecosystem National Observation and Research Station, Leshan, China; 4 Ganzi Institute of Forestry Research, Kangding, China; 5 Gateway Antarctica, Centre for Antarctic Studies and Research, School of Earth & Environment, University of Canterbury, Christchurch, New Zealand; 6 Department of Plant Pathology, College of Plant Protection, China Agricultural University, Beijing, China; 7 Bamboo Resource Conservation and Utilization Key Laboratory of Sichuan Province, Leshan, China

**Keywords:** classification, diversity, macrofungi, phylogenetic analysis, wood-rotting fungi

## Abstract

During a survey of wood-decaying fungi in the subalpine forests of Southwest China, distinctive corticioid specimens were collected from fallen *Abies* trunks. Based on a combination of morphological characteristics and multigene phylogenetic analyses of the internal transcribed spacer (ITS) and nuclear large subunit (nLSU) sequences, these specimens were found to represent a highly distinct and strongly supported monophyletic lineage within the family Hydnaceae (Order: Cantharellales; Division: Basidiomycota). Consequently, a new genus, *Leucopruina* gen. nov., is proposed to accommodate the novel species *L. abieticola* sp. nov. Morphologically, the new species is characterized by its white, pruinose, resupinate basidiomata and a dimitic hyphal system with branched skeletal hyphae. Although it shares urniform basidia bearing 6–8 sterigmata with *Sistotremella*, *Leucopruina* is clearly distinguished by its thin-walled, acyanophilous (CB–) basidiospores, in contrast to the thick-walled, cyanophilous (CB+) spores of *Sistotremella*. This discovery increases the number of recognized genera within Hydnaceae to 19 and highlights the rich but previously underestimated taxonomic diversity of saprotrophic fungi in the high-altitude ecosystems of Southwest China.

## Introduction

The family Hydnaceae Chevall., originally established for fungi with hydnoid hymenophores similar to the type genus *Hydnum* L. (Miller, 1933), is currently classified within the order Cantharellales, where it forms a nested clade with Botryobasidiaceae, Ceratobasidiaceae, and Tulasnellaceae (Hibbett et al., 2014). Recent multilocus phylogenetic analyses (based on 28S, ITS, mtSSU, *rpb2*, and *tef-1α*) have significantly refined the family boundaries, resolving it as a robust and diverse clade within the order Cantharellales (Cao et al., 2021). According to the latest taxonomic integration by He et al. (2024), the family currently encompasses 18 recognized genera, including *Bergerella* Diederich & Lawrey, *Bryoclavula* H. Masumoto & Y. Degawa, *Bulbilla* Diederich, Flakus & Etayo, *Burgella* Diederich & Lawrey, *Burgellopsis* Diederich & Lawrey, *Burgoa* Goid, *Cantharellus* Adans. ex Fr., *Clavulina* J. Schröt., *Craterellus* Pers., *Hydnum*, *Membranomyces* Jülich, *Minimedusa* Weresub & P. M. LeClair, *Multiclavula* R. H. Petersen, *Neoburgoa* Diederich, E. Zimm. & Lawrey, *Parmeliicida* Diederich, F. Berger, Etayo & Lawrey, *Rogersiomyces* J. L. Crane & Schokn, *Sistotrema* J. L. Crane & Schokn., and *Sistotremella* Hjortstam.

Despite this clear phylogenetic delimitation, the Hydnaceae remain exceptionally diverse in their morphological characters. Its members exhibit a wide array of basidiocarp forms—ranging from cantharelloid (e.g., *Cantharellus* and *Craterellus*) and clavarioid (e.g., *Clavulina* and *Multiclavula*) to corticioid (e.g., *Sistotrema* and *Membranomyces*)—and diverse hymenophore structures, including hydnoid (e.g., *Hydnum*), poroid (e.g., *Sistotrema*), smooth (e.g., *Cantharellus*), or veined configurations (e.g., *Craterellus*). Furthermore, the number of sterigmata per basidium shows remarkable intergeneric variation, typically ranging from two (e.g., *Clavulina* and *Membranomyces*), two to six (e.g., *Cantharellus*), to as many as eight (e.g., *Sistotrema* and *Sistotremella*) (Cao et al., 2021; Zhou et al., 2025). Moreover, many members are well-known culinary mushrooms (e.g., *Cantharellus*, *Clavulina*, *Craterellus,* and *Hydnum*) (Boa, 2004; Dai et al., 2010; Wu et al., 2019).

However, taxonomic gaps persist within the family. While most genera are monophyletic, *Sistotrema* is recognized as highly polyphyletic (Moncalvo et al., 2006; Nilsson et al., 2006; Larsson, 2007; Veldre et al., 2013; Hibbett et al., 2014), suggesting that the true generic diversity of Hydnaceae remains underestimated.

Previously, more than 20 new species of wood-inhabiting fungi have been described from Sichuan Province (Dai et al., 2004, 2021; Yuan et al., 2021), reflecting the province’s rich diversity of woody plants and its potential to harbor additional undescribed species. During our survey of wood-decaying fungi in Southwest China, two distinctive corticioid specimens were collected from fallen *Abies* trunks. Molecular phylogenetic analyses (based on ITS + nLSU sequences) revealed that these specimens represent a stable and independent monophyletic lineage within the family Hydnaceae. This lineage exhibits significant genetic distance from all currently recognized genera, with ITS sequence similarities falling well below the typical thresholds for congenericity. Combined with its consistent morphological divergence from closely related taxa, this discovery provides strong evidence for the establishment of a new genus. Consequently, the novel genus *Leucopruina* gen. nov., typified by *L. abieticola* sp. nov., is proposed herein to accommodate this unique lineage.

## Materials and methods

### Collections

The fungal materials were collected from Sichuan Province, China. For each specimen, we recorded the specific habitat, collection date, and exact locality and deposited the voucher specimens in the Fungarium of Sichuan Agricultural University (SAUFC, China). Notably, the holotype was discovered in the Miyaluo Scenic Area, Li County, Aba, Sichuan Province, on a fallen trunk of *Abies* (102°38′E, 31°37′N, altitude 3544.5 m). This area is characterized by subalpine primitive forests dominated by *Picea asperata* Mast., *Abies fabri* (Mast.) Craib, and *Betula platyphylla* Sukaczev. The detailed information for the specimens examined is as follows: 26 August 2025, Z. B. Liu 669 (holotype, SAUFC 000669); 31 August 2025, Z. B. Liu 316 (paratype, SAUFC 000316).

### Morphological studies

Macromorphological descriptions were based on field notes and dry herbarium specimens. Microscopic structures were examined in slides prepared with dried tissues using an Olympus BX43 compound microscope at 1,000 × magnification. Measurements were usually taken from sections mounted in Cotton Blue or Melzer’s reagent. The following abbreviations were used: KOH = 5% potassium hydroxide; L = mean spore length (the arithmetic mean of all measured spores); W = mean spore width (the arithmetic mean of measured spores); Q = variation in the L/W ratios among the specimens examined; and *n* (a/b) = number of spores (a) measured from a given number of specimens (b). In presenting spore size variation, 5% of measurements at both ends of the range were excluded, and the extreme values were given in parentheses. Special color terms follow Petersen (1996). Herbarium abbreviations follow Thiers (2018).

### DNA extraction, PCR amplification, and sequencing

Total genomic DNA was extracted from dried specimens using a cetyltrimethylammonium bromide (CTAB) rapid plant genome extraction kit (Aidlab Biotechnologies Company, Limited, Beijing, China) according to manufacturer’s instructions, with some modifications (following Dong et al., 2024; Yang et al., 2025). The ITS region was amplified with primers ITS5 and ITS4 (White et al., 1990), whereas the nLSU region was amplified with primers LR0R and LR7 (Vilgalys and Hester, 1990).

The polymerase chain reaction (PCR) procedure for ITS was as follows: initial denaturation at 95 °C for 3 min, followed by 35 cycles at 94 °C for 40 s, 58 °C for 45 s, and 72 °C for 1 min, and a final extension of 72 °C for 10 min (Dong et al., 2025). The PCR procedure for nLSU was as follows: initial denaturation at 94 °C for 1 min, followed by 35 cycles at 94 °C for 30 s, 48 °C for 1 min, and 72 °C for 1.5 min, and a final extension of 72 °C for 10 min (Deng et al., 2026). The PCR products were purified and sequenced by Sangon Biotech (Shanghai) Co., Ltd. (Chengdu, China) using the same primers as those used in the PCR reactions. The NCBI database was searched for homologous sequences of related species using the BLAST program (https://blast.ncbi.nlm.nih.gov).

### Phylogenetic analyses

**Figure 1:**
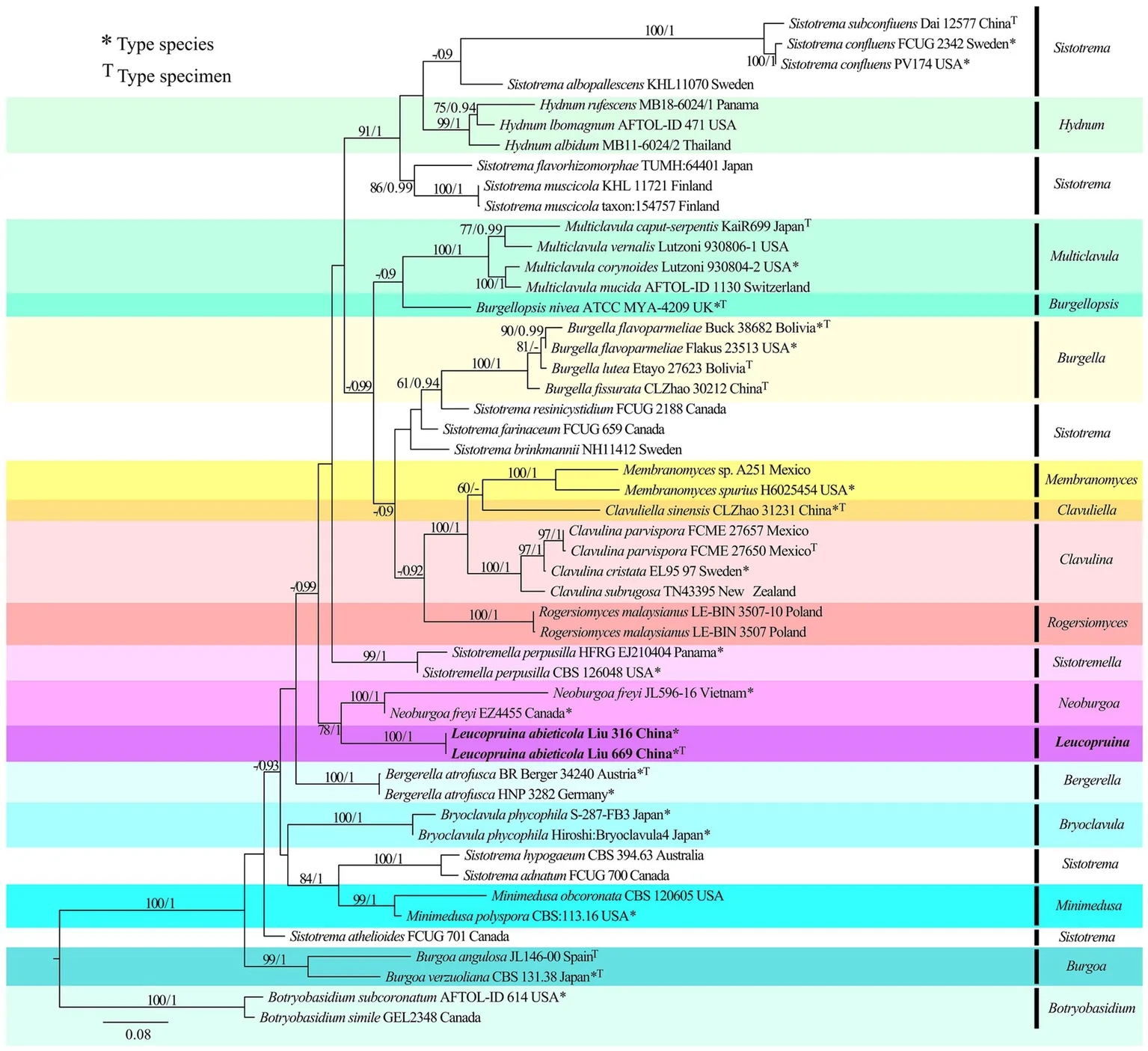
Phylogeny of Hydnaceae and related species generated by ML analysis based on combined ITS + nLSU sequences. Branches are labeled with maximum likelihood bootstrap support values >60% and Bayesian posterior probabilities >0.90. The new genus and species are shown in bold. ITS, internal transcribed spacer; ML, maximum likelihood; nLSU, nuclear large subunit.

**Figure 2:**
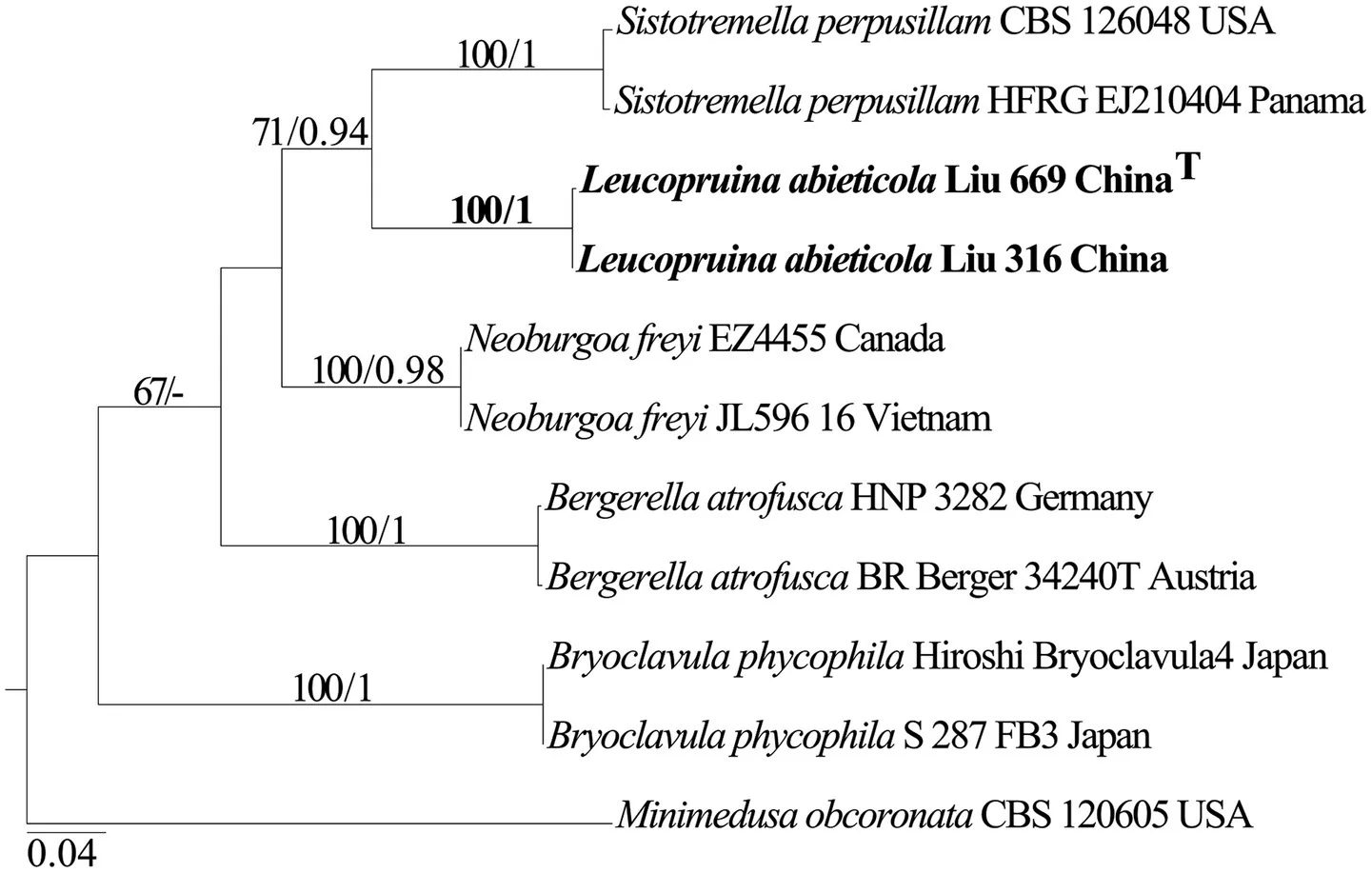
Phylogeny of *Leucopruina* and related species generated by ML analysis based on ITS sequences. Branches are labeled with maximum likelihood bootstrap support values >60% and Bayesian posterior probabilities >0.90. The new genus and new species are shown in bold. ITS, internal transcribed spacer; ML, maximum likelihood.

Phylogenetic trees were constructed using ITS and ITS + nLSU rDNA sequences, and phylogenetic analyses were performed using maximum likelihood (ML) and Bayesian inference (BI) methods. Sequences of the species and strains were selected primarily based on the ITS + nLSU tree topology described in the study of Zhou et al. (2025). New sequences generated in this study, along with reference sequences retrieved from GenBank, were aligned using MAFFT v7 (Katoh et al., 2019; http://mafft.cbrc.jp/alignment/server/) under the “G-INS-i” strategy and manually adjusted in BioEdit (Hall, 1999). Unreliably aligned sections were manually inspected and excluded prior to phylogenetic analyses. The data matrix was edited in Mesquite v3.81 software (Maddison and Maddison, 2023). The sequence alignments were deposited in Figshare (10.6084/m9.figshare.32306262, Figure 1, 10.6084m9.figshare.32880134, Figure 2). Sequences of *Botryobasidium subcoronatum* (Höhn. & Litsch.) Donk and *Botryobasidium simile* Pouzar & Hol.-Jech. obtained from GenBank were used as outgroups to root the trees in the ITS + nLSU analyses (Table 1).

**Table 1:** Information on the taxa used in phylogenetic analyses.

Species name	Specimen No.	GenBank accession No.		Country
		ITS	nLSU	
Bergerella atrofusca*	BR Berger 34240^T^	MN902070	MN902070	Austria
Bergerella atrofusca*	HNP 3282	PX651235	—	Germany
*Botryobasidium simile*	GEL2348	KP171641	DQ898730	Canada
Botryobasidium subcoronatum*	AFTOL-ID 614	DQ200924	AY647212	USA
*Bryoclavula phycophila*	S-287-FB3	LC544109	—	Japan
Bryoclavula phycophila*	Hiroshi:Bryoclavula4	OQ791465	OQ791464	Japan
*Burgella fissurata*	CLZhao 30212^T^	PQ758749	PQ758757	China
*Burgella flavoparmeliae*	Buck 38682^T^	—	DQ915469	Bolivia
Burgella flavoparmeliae*	Flakus 23513	—	KC336074	USA
*Burgella lutea*	Etayo 27623^T^	KC336076	KC336075	Bolivia
Burgellopsis nivea*	ATCC MYA-4209^T^	—	KC336077	UK
*Burgoa angulosa*	JL146-00^T^	—	DQ915471	Spain
Burgoa verzuoliana*	CBS 131.38^T^	NR145334	NG058614	Japan
Clavuliella sinensis*	CLZhao 31231^T^	PQ758750	PQ758758	China
Clavulina cristata*	EL95_97	—	AY586648	Sweden
*Clavulina parvispora*	FCME 27650^T^	MH542550	MN049492	Mexico
*Clavulina parvispora*	FCME 27657	MH542549	MN049491	Mexico
*Clavulina subrugosa*	TN43395	JN228221	JN228221	New Zealand
*Hydnum albidum*	MB11-6024/2	—	AY293186	Thailand
*Hydnum albomagnum*	AFTOL-ID 471	DQ218305	AY700199	USA
*Hydnum rufescens*	MB18-6024/1	—	AY293187	Panama
Leucopruina abieticola*	**Liu 669** ^**T**^	**PZ428759**	—	**China**
Leucopruina abieticola*	**Liu 316**	**PZ428760**	—	**China**
*Membranomyces* sp.	A251	PV766500	—	Mexico
Membranomyces spurius*	H6025454	JQ638713	—	USA
*Minimedusa obcoronata*	CBS 120605	GQ303278	GQ303309	USA
Minimedusa polyspora*	CBS:113.16	—	MH866167	USA
*Multiclavula caput-serpentis*	KaiR699^T^	MW386064	MW369074	Japan
Multiclavula corynoides*	Lutzoni 930804-2	U66440	U66440	USA
*Multiclavula mucida*	AFTOL-ID 1130	DQ521417	AY885163	Switzerland
*Multiclavula vernalis*	Lutzoni 930806-1	U66439	U66439	USA
Neoburgoa freyi*	JL596-16	KX423755	KX423755	Vietnam
Neoburgoa freyi*	EZ4455	OR471314	OR471068	Canada
*Rogersiomyces malaysianus*	LE-BIN 3507-10	KT779285	KU820986	Poland
*Rogersiomyces malaysianus*	LE-BIN 3507	KT779284	KT779286	Poland
*Sistotrema adnatum*	FCUG 700	OR464426	OR460895	Canada
*Sistotrema albopallescens*	KHL11070	—	AM259210	Sweden
*Sistotrema athelioides*	FCUG 701	—	DQ898700	Canada
*Sistotrema brinkmannii*	NH11412	—	AF506473	Sweden
Sistotrema confluens*	FCUG298	DQ267125	AY647214	USA
Sistotrema confluens*	PV174	AY463466	AY586712	Sweden
*Sistotrema farinaceum*	FCUG 659	—	DQ898707	Canada
*Sistotrema flavorhizomorphae*	TUMH:64401	LC642038	LC642059	Japan
*Sistotrema hypogaeum*	CBS 394.63	MH858314	MH869926	Australia
*Sistotrema muscicola*	taxon:154757	AJ606041	AJ606041	Finland
*Sistotrema muscicola*	KHL 11721	AJ606040	AJ606040	Finland
*Sistotrema resinicystidium*	FCUG 2188	—	DQ898708	Canada
*Sistotrema subconfluens*	Dai 12577^T^	JX076812	JX076810	China
Sistotremella perpusilla*	CBS 126048	MH864061	MH875516	USA
Sistotremella perpusilla*	HFRG EJ210404	OL828790	—	Panama

*Indicates the type species, and ^T^indicates the type specimen. The new genus and species are shown in bold. ITS, internal transcribed spacer; nLSU, nuclear large subunit.

The ML analysis was conducted using RAxML-HPC v. 8.2.3 (Stamatakis, 2014) implemented through the CIPRES Science Gateway (Miller et al., 2009; http://www.phylo.org). Statistical support values (BS) were obtained using non-parametric bootstrapping with 1,000 replicates. The BI analysis was performed with MrBayes 3.2.7a (Ronquist and Huelsenbeck, 2003). Two runs, each with four Markov chains starting from random trees, were performed for 6 million generations for the dataset in Figure 1 and 0.4 million generations for the dataset in Figure 2 until the average standard deviation of split frequencies fell below 0.01; trees were sampled every 1,000 generations. The first 25% of the sampled trees were discarded as burn-in, and the remaining ones were used to reconstruct a majority-rule consensus tree and calculate Bayesian posterior probabilities (BPP) of the clades.

The optimal substitution models for the datasets were determined using the Akaike Information Criterion (AIC) implemented in MrModeltest 2.3 (Posada and Crandall, 1998; Nylander, 2004) after evaluating 24 models of nucleotide evolution in PAUP* version 4.0 beta 10 (Swofford, 2002). The models selected for Bayesian inference (BI) were GTR + F + I + G4 for the dataset in Figure 1 and HKY + F + G4 for the dataset in Figure 2.

Branches with maximum likelihood (ML) bootstrap support (BS) > 60% and Bayesian posterior probabilities (BPP) > 0.9 were considered significantly supported. The ML analysis produced the best-scoring tree, which is presented with both ML bootstrap support values and BI analyses. FigTree v1.4.4 (Rambaut, 2012) was used to visualize the resulting tree.

## Results

### Phylogenetic analyses

The concatenated ITS + nLSU dataset contained sequences from 50 fungal specimens representing 43 Hydnaceae taxa. The dataset had an aligned length of 1,855 characters, of which 966 were constant, 181 were variable but parsimony-uninformative, and 708 were parsimony-informative. The best-fit model for the ITS + nLSU dataset used in the BI analysis was GTR + F + I + G4. The average standard deviation of split frequencies at the end of the Bayesian analysis was 0.003501 (BI), and the effective sample size (ESS) values for the Bayesian analysis across the two runs were double the average ESS (avg ESS = 3,529). The phylogenetic tree inferred from the ITS + nLSU sequences showed that the new genus *Leucopruina* clustered within the family Hydnaceae (Figure 1).

The ITS dataset contained sequences from 11 fungal specimens representing six Hydnaceae taxa. The dataset had an aligned length of 846 characters, of which 557 were constant, 81 were variable but parsimony-uninformative, and 208 were parsimony-informative. The best model for the ITS dataset in the BI analysis was HKY + F + G4. The average standard deviation of split frequencies in the Bayesian analyses was 0.007752 (BI), and the effective sample size (ESS) for the Bayesian analysis across the two runs was double the average ESS (avg ESS = 345). The topology based on the ITS dataset showed that *Leucopruina* formed an independent lineage most closely allied with *Sistotremella* (Figure 2).

### Taxonomy

**Leucopruina** J. L. Zhang, K. N. Meng & Z. B. Liu, **gen. nov**.

Type species: *Leucopruina abieticola* J. L. Zhang, K. N. Meng & Z. B. Liu, sp. nov.

MycoBank number: MB 863794.

Etymology: *Leucopruina* (Latin): *Leuco- is derived* from the Greek *leukos*, meaning “white”; whereas *-pruina* is derived from Latin, meaning “hoar-frost” or “fine powder.” The name refers to the characteristic white, pruinose hymenial surface of the basidiomata.

Habitat: Growing on fallen trunks of *Abies*.

Description: Basidiomata annual, resupinate, closely adnate, inseparable from the substrate, without odor or taste when fresh. Hymenial surface smooth and pruinose, white when fresh, becoming buff when dry; uncracked in both the fresh or dry states. Sterile margin indistinct. Hyphal system dimitic; generative hyphae bearing clamp connections, hyaline, thin-walled, occasionally branched; skeletal hyphae dominant, hyaline, thick-walled, usually branched, interwoven; all hyphae IKI–, CB–. Tissues dissolved in KOH. Cystidia and cystidioles absent; basidia clavate to urniform, with 6–8 sterigmata and a basal clamp connection; basidioles similar in shape to but smaller than basidia. Basidiospores ellipsoid, hyaline, thin-walled, smooth, usually with guttules, IKI–, CB–.

**Figure 3:**
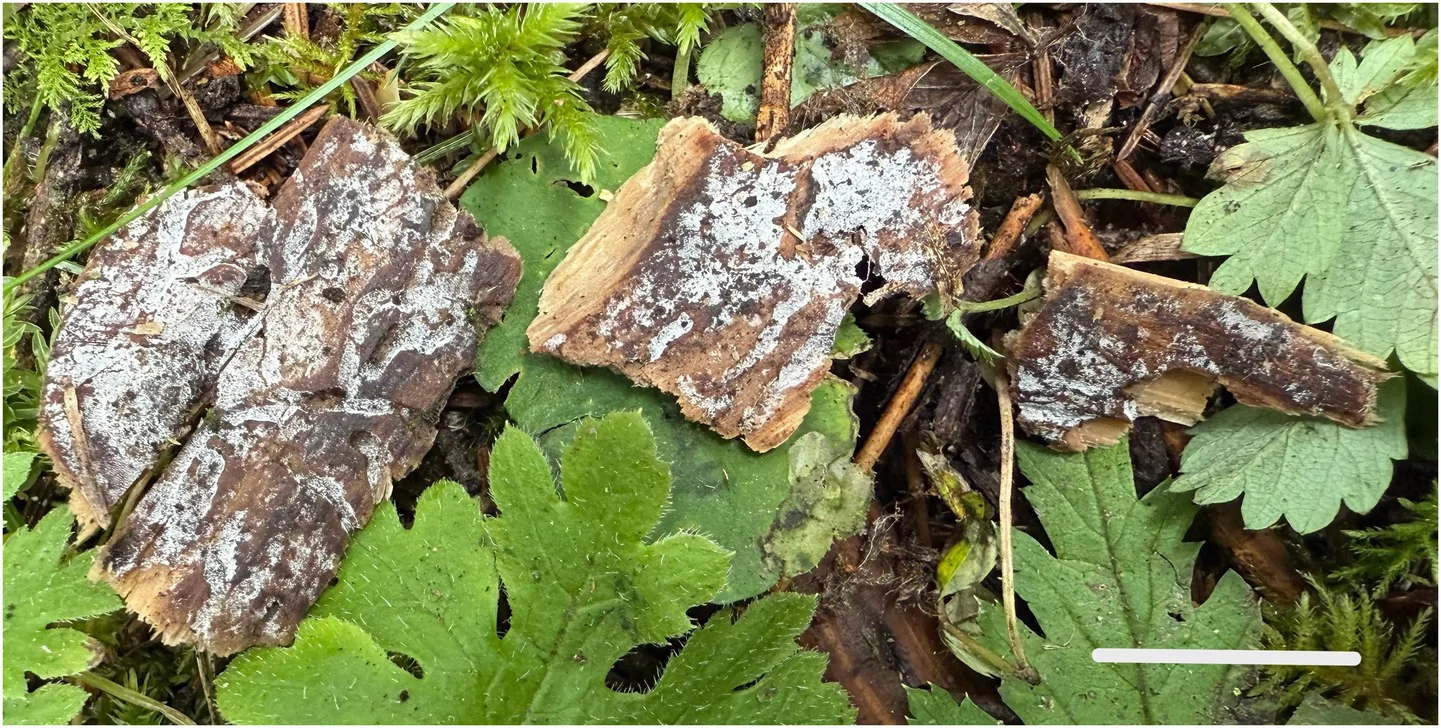
Basidiomata of *Leucopruina abieticola* (holotype, Liu 669). Scale bar = 1.0 cm. Photograph by Zhan-Bo Liu.

**Figure 4:**
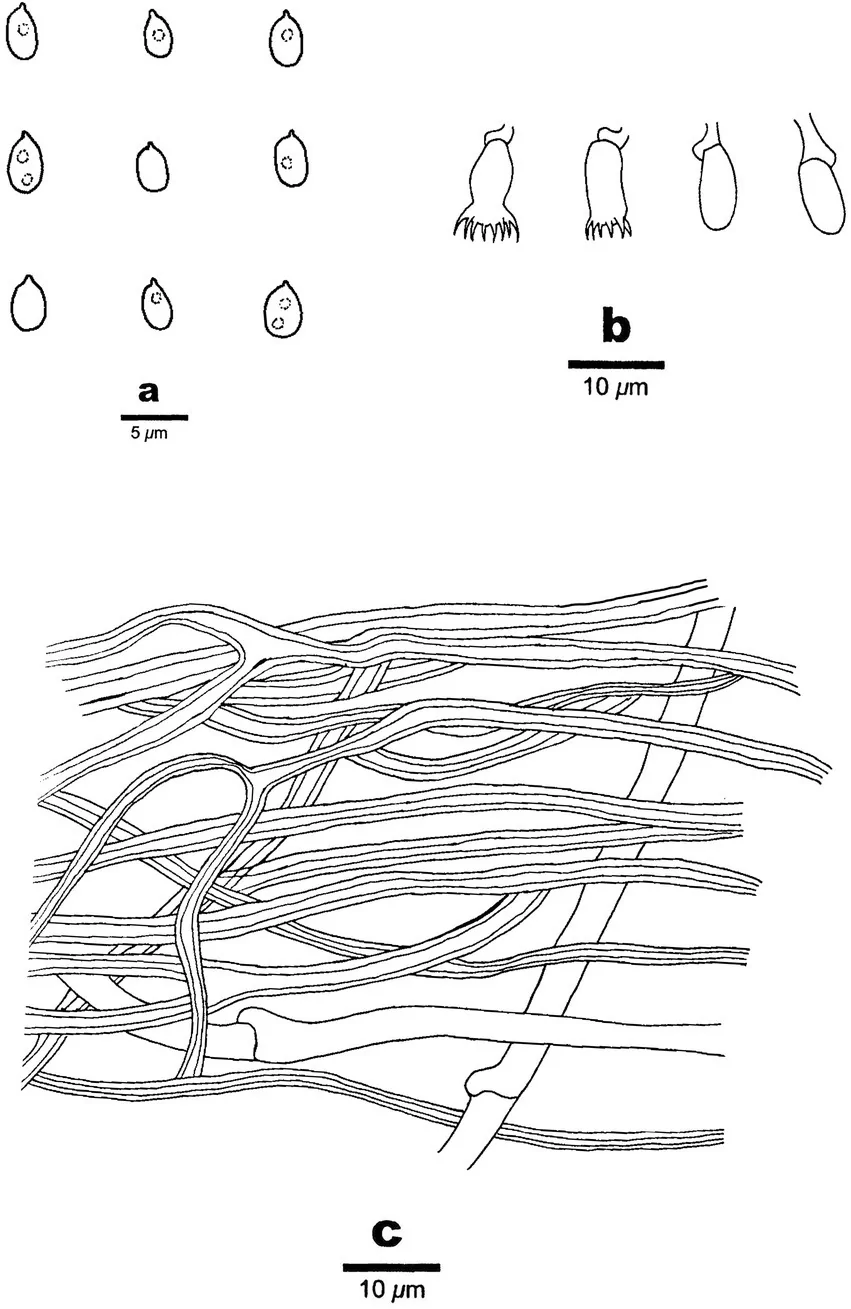
Microscopic structures of *Leucopruina abieticola* (holotype, Liu 669). **(a)** Basidiospores. **(b)** Basidia and basidioles. **(c)** Hyphae in the subhymenium. Drawings by Zhan-Bo Liu.

**Leucopruina abieticola** J. L. Zhang, K. N. Meng & Z. B. Liu, **sp. nov.** Figures 3, 4.

MycoBank number: MB 863806.

Type: China, Sichuan Province, Aba, Li County, Miyaluo Scenic Area, on a fallen trunk of *Abies*, in southwestern China, ca. E 102° 38′, N 31° 37′, alt. 3544.5 m. The vegetation consists of a subalpine primitive forest dominated by *Picea asperata* Mast., *Abies fabri* (Mast.) Craib, and *Betula platyphylla* Sukaczev. 26 August 2025, Z. B. Liu 669 (holotype, SAUFC 000669).

Etymology: *Abieticola* (Latin) refers to the species growing on the wood of *Abies*.

Description: Basidiomata annual, resupinate, closely adnate, inseparable from the substrate, growing on the underside of the trunk, without odor or taste when fresh, up to 2 cm long, 1 cm wide, and 0.3 mm thick. Hymenial surface smooth and pruinose, white when fresh, becoming buff when dry; uncracked when fresh or dry. Sterile margin indistinct.

Hyphal structure: Hyphal system dimitic; generative hyphae with clamp connections, hyaline, thin-walled, occasionally branched, 2–5.5 μm in diameter; skeletal hyphae dominant, hyaline, thick-walled, usually branched, interwoven, 2–4.5 μm in diameter; all hyphae IKI–, CB–. Tissues dissolved in KOH.

Hymenium: Cystidia and cystidioles absent; basidia clavate to urniform, with 6–8 sterigmata and a basal clamp connection, 11.5–12.5 × 4–6 μm; basidioles similar in shape to, but smaller than, basidia.

Basidiospores: Ellipsoid, hyaline, thin-walled, smooth, usually with guttules, IKI–, CB–, (3.4–)3.5–5.4 × (2.2–)2.3–3.4(−3.7) μm, L = 4.13 μm, W = 2.7 μm, Q = 1.46–1.57 (*n* = 60/2).

Additional specimen examined (paratype): China, Sichuan Province, Aba, Li County, Miyaluo Scenic Area, on a fallen trunk of *Abies*, 31 August 2025, Z. B. Liu 316 (SAUFC 000316).

## Discussion

The present study introduces *Leucopruina* as a novel genus within Hydnaceae, a placement robustly supported by its distinct morphological characteristics and multigene phylogenetic analyses. The phylogenetic placement of *Leucopruina* within Hydnaceae was resolved through a two-stage analytical approach. Initially, a multigene (ITS + nLSU) framework was constructed using available sequences from GenBank to identify the broader taxonomic neighborhood of the new genus (Figure 1). Although we successfully integrated nLSU data for previously described taxa, persistent amplification and sequencing contamination issues specific to the new genus prevented us from obtaining a high-quality nLSU sequence from our specimens. Consequently, the multigene analysis for *Leucopruina* relied solely on the ITS region. Nevertheless, this multigene framework proved essential for selecting a representative, closely related set of operational taxonomic units (OTUs) for a refined phylogenetic analysis.

Subsequently, we conducted an objective, ITS-only phylogenetic analysis using this narrow set of species (Figure 2), which allowed for a high-quality, unambiguous sequence alignment—an outcome that would have been statistically compromised had we included the entire, more distant ITS dataset. This refined analysis robustly positions *Leucopruina* as an independent lineage most closely allied with *Sistotremella* (Figure 2) rather than supporting the sister relationship with *Neoburgoa*, as suggested by the broader multigene tree (Figure 1). The profound genetic distance observed in the ITS region (>11% divergence) exceeds typical congeneric thresholds within the family Hydnaceae, providing strong molecular evidence for the distinct generic status of *Leucopruina*. These results, combined with the clear topological separation, warrant the establishment of *Leucopruina* as a distinct genus, thereby increasing the total number of recognized genera in the family to 19.

The differences between *Leucopruina* and its closest relatives, *Sistotremella* and *Neoburgoa*, are substantial and involve both ecological and morphological disparities. Although *Leucopruina* and *Sistotremella* share a similar resupinate habit and the diagnostic urniform basidia bearing 6–8 sterigmata—a character also found in the white resupinate lineages of the polyphyletic *Sistotrema*—they can be readily segregated by fundamental differences in hyphal construction and spore chemistry. *Leucopruina* is characterized by a unique dimitic hyphal system with branched skeletal hyphae, a structural complexity highly unusual among corticioid members of Hydnaceae. In contrast, both *Sistotremella* and the white resupinate lineages of *Sistotrema* are strictly monomitic, consisting only of generative hyphae with clamps (Cao et al., 2021). Furthermore, while *Sistotremella* possesses distinctly thick-walled, strongly cyanophilous (CB+) basidiospores, *Leucopruina* exhibits thin-walled, acyanophilous (CB–) spores. At the species level, the basidiospores of *L. abieticola* are larger than those of the type species *Sistotremella perpusilla* (3.5–5.4 × 2.3–3.4 μm vs. 3–5 × 2–2.5 μm, Eriksson et al., 1984).

The evolutionary divergence is further reinforced by their distinct life-history strategies. Although *Leucopruina* is phylogenetically allied with *Sistotremella* and maintains a sister relationship to *Neoburgoa* in the broader multigene framework, these genera occupy disparate ecological niches. *Neoburgoa* is a highly specialized lichenicolous genus that grows exclusively on the thalli of *Cladonia rangiferina* (L.) F. H. Wigg., produces pale yellow to orange bulbils, and lacks known basidiomata or conidiomata (Lawrey et al., 2016). In contrast, *Leucopruina* is a saprotrophic, wood-decaying fungus that develops well-developed annual, resupinate basidiomata with a white, pruinose hymenial surface. This transition—from the lichen-associated, bulbil-forming anamorph of *Neoburgoa* to the wood-inhabiting, sexually reproducing corticioid habit of *Leucopruina*—further justifies their separation at the generic level.

The discovery of *Leucopruina abieticola* on fallen trunks of *Abies* in Southwest China underscores the exceptional fungal diversity of this region. Southwest China, particularly the subalpine forests of Sichuan, is recognized as a global biodiversity hotspot for wood-decaying fungi (Dong et al., 2024). The presence of a specialized, dimitic corticioid lineage on coniferous wood suggests that the taxonomic and functional diversity of saprotrophic Hydnaceae in high-altitude ecosystems remains underestimated, and further exploration of these specialized niches is warranted.

Wood-inhabiting fungi have been extensively studied in China, and several new genera and families have been proposed (Wu et al., 2004; Zhao et al., 2015; Wu et al., 2022; Zhou et al., 2023, 2026). Our present study confirms that additional high-level macrofungal taxa remain to be discovered.

## Data Availability

The sequence alignment is deposited at Figshare (https://doi.org/10.6084/m9.figshare.32306262, Figure 1; https://doi.org/10.6084m9.figshare.32880134, Figure 2).
